# Editorial: Propriospinal Neurons: Essential Elements in Locomotion, Autonomic Function and Plasticity After Spinal Cord Injury and Disease

**DOI:** 10.3389/fncel.2021.695424

**Published:** 2021-05-20

**Authors:** Kristine C. Cowley, Michael A. Lane, Claire F. Meehan, Michelle M. Rank, Katinka Stecina

**Affiliations:** ^1^Department of Physiology and Pathophysiology, University of Manitoba, Winnipeg, MB, Canada; ^2^Spinal Cord Research Centre, Max Rady College of Medicine, University of Manitoba, Winnipeg, MB, Canada; ^3^Department of Neurobiology and Anatomy, College of Medicine, Drexel University, Philadelphia, PA, United States; ^4^Marion Murray Spinal Cord Research Centre, Drexel University, Philadelphia, PA, United States; ^5^Department of Neuroscience, Faculty of Health Sciences, University of Copenhagen, Copenhagen, Denmark; ^6^Department of Anatomy and Physiology, The University of Melbourne, Parkville, VIC, Australia

**Keywords:** propriospinal, neuron, respiration, locomotion, autonomic function, motor control, central pattern generator

Propriospinal, the combination of a Latin expression for “one's own” and the word spinal, refers to neurons with somas and axons terminating within the extent of the spinal cord. Propriospinal neurons (PSNs) are contained entirely within the spinal cord and may have short segmental or multi-segment projections. Seminal research on PSNs lead by Lundberg (1979) first identified separate populations of PSNs: with either short- (1–3 spinal segments) (Jankowska et al., 1974, 1983; Edgley and Jankowska, 1987) or long-distance projections (spanning several spinal segments) (Jankowska et al., 1974). A well-recognized example of the latter are the cervical PSNs directly coordinating forelimb-hindlimb function (Alstermark et al., 1979). This early research underpins the more modern discoveries detailing the functional properties of anatomically distinct PSN populations. This series of articles uses the broadest definition of PSNs with an emphasis on operational capacity in order to highlight the full integrative capacity of PSNs. In fact, the complex integration capabilities of all PSN subpopulations were recognized in early research stages. Experiments using decerebrate cat preparations allowed for the simultaneous examination of inputs from multiple brain regions (Illert and Lundberg, 1978) and from peripheral sources to PSNs, thus highlighting the core integration functions of PSNs. While significant advances have been gained in research capabilities since this early work, the theme of integration has remained as a stronghold.

Propriospinal neurons can convey descending commands (Cowley et al., 2008) and participate in the integration of these commands with afferent sensory feedback from the periphery. They also serve to mediate and coordinate rhythmic motor output involving multiple joints, with corresponding neurons spanning several spinal segments. Discrete networks of PSNs constitute essential building blocks of the central pattern generators for respiration, locomotion and autonomic functions. Three in depth review articles in this collection revisit decades of research to provide a new perspective on the current understanding of the role of PSNs in these research fields. Laliberte et al. provide a broad-spectrum analysis of evidence that PSNs are not only acting as essential elements of locomotor control circuits in the intact spinal cord, but also play a significant role after spinal cord injury. Respiratory PSNs have been described in the spinal cord of many species as discussed by Jensen et al. and these PSN populations contribute to the recovery of breathing after spinal cord injury. An excellent overview on how plasticity of PSNs is associated with the recovery of autonomic function is given by Michael et al. in relation to complete spinal injury and autonomic dysreflexia.

Given the complexity of PSNs and their diverse functional capabilities, how might we dissect these propriospinal circuits? Developmental markers to identify or manipulate specific classes of propriospinal neurons offer valuable tools to investigate spinal circuits. While knowledge about PSN-motoneuron connectivity has advanced substantially with the help of molecular, genetic, anatomical, and electrophysiological research techniques; mapping the landscape of spinal interneuronal connectivity has lagged behind. Experimental approaches addressing this gap are offered by Haque and Gosgnach.

The analysis of locomotor circuits has especially benefited from developmental genetics. In this collection, three genetically defined populations, the so-called V3, Shox2, and Hb9 interneurons have been examined by Danner et al. Here they make innovative use of murine optogenetic approaches and combine these results with large-scale computational modeling studies to show that V3 commissural neurons provide mutual excitation to separate populations of neurons involved in left-right extensor activity. The experiments by Li et al. show that Shox2 neurons are interposed in multiple sensory pathways. Furthermore, these Shox2 neurons, receiving input from low threshold proprioceptive flexor and extensor nerves, can activate or inhibit both the rhythm and patterning layers of the locomotor central pattern generator. Buntschu et al. combined patch-clamp and array recordings to investigate Hb9 interneurons in spinal cord cultures. Their findings described key ion channels that contribute to spontaneous intrinsic and repetitive spiking and thereby to the generation of network bursts.

Develle and Leblond examine the pharmacological manipulations of hindlimb reflexes within hours after spinal injury. Unexpected biphasic effects on PSNs of segmental reflex loops were identified by applying serotonin receptor agonists known to trigger stepping in mice after complete spinal transections.

Combinatorial strategies targeting PSN networks to promote recovery after spinal cord injury were addressed in two studies. A study by Mahrous et al. utilized combined stimulation of sensory and motor inputs (electrically or pharmacologically) that lead to more stable motor output than sensory or motor stimulation alone. The experiment by Chia et al. evaluated spontaneous stepping freely performed by rats. The methods and algorithms developed by this work provide exciting possibilities for examining the PSN contribution to free movement in the home-cage environment.

In addition to locomotion, postural reflexes are also dependent on PSNs. Zelenin et al. showed that PSNs undergo rapid changes within days of spinalization that allows for the restoration of normal activity levels in spinal interneurons, and a slow recovery over months that restores motoneurone excitability. Postural responses, however, fail to be reinstated and poor sensory-motor coordination of timing remains.

Peyre et al. investigated the effects of listening to music, albeit arrhythmic music, on walking in stroke survivors. They showed that a knee excitation reflex evoked by pre-tibial flexor stimulation after a stroke was significantly larger during walking without sound than when listening to music. This suggests that arrhythmic music listening modulates propriospinal excitability during post-stroke walking; likely representing brainstem modulation of PSN activity. In other words, music may be useful to “retrain” sensory-motor coordination via PSN networks after a neural injury in humans.

In summary, the articles brought together in this special edition highlight fundamental work on PSNs and the networks contributing to motor and autonomic functions, and do so from a broad perspective, reflecting the diverse role of PSNs. The review papers in this collection reinforce the critical role of sensory input in the recruitment of PSNs as well as in directing the output of PSN networks. An additional important finding reflected in the original research papers is that PSNs play a prominent and essential role in both hindlimb and forelimb coordination and respiratory muscle activity—in both health and disease. The research collected here reminds us that little is currently understood about the role of PSNs in autonomic functions under normal or abnormal conditions. This special collection of research is both fascinating and exciting, and overall suggests that it is finally time to shift the paradigm on PSNs. Rather than viewing PSNs as a uniform neuronal network with limited functional impact, it is now clear that they are in fact a diverse population of neurons. PSNs are critical components of a variety of networks with important functional roles beyond mere limb coordination.

## Author Contributions

All authors listed have made a substantial, direct and intellectual contribution to the work, and approved it for publication.

## Conflict of Interest

The authors declare that the research was conducted in the absence of any commercial or financial relationships that could be construed as a potential conflict of interest.
